# Pragmatic RAndomised controlled trial of a trauma-focused guided self-help Programme versus InDividual trauma-focused cognitive Behavioural therapy for post-traumatic stress disorder (RAPID): trial protocol

**DOI:** 10.1186/s12888-018-1665-3

**Published:** 2018-03-27

**Authors:** Claire Nollett, Catrin Lewis, Neil Kitchiner, Neil Roberts, Katy Addison, Lucy Brookes-Howell, Sarah Cosgrove, Katherine Cullen, Anke Ehlers, Sarah Heke, Mark Kelson, Karina Lovell, Kim Madden, Kirsten McEwan, Rachel McNamara, Ceri Phillips, Timothy Pickles, Natalie Simon, Jonathan Bisson

**Affiliations:** 1 Centre for Trials Research, Cardiff University, 4th Floor Neuadd Meirionnydd, Heath Park, Cardiff, CF14 4YS UK; 2 Division of Psychological Medicine and Clinical Neurosciences, Cardiff University School of Medicine, Haydn Ellis Building, Maindy Road, Cardiff, CF24 4HQ UK; 3 Cardiff & Vale University Health Board, Cardiff University School of Medicine, Haydn Ellis Building, Maindy Road, Cardiff, CF24 4HQ UK; 4 Centre for Trials Research, Cardiff University, 7th Floor Neuadd Meirionnydd, Heath Park, Cardiff, CF14 4YS UK; 5 Independent Public and Patient Involvement (PPI) representative, Cardiff, UK; 6 Swansea Centre for Health Economics, College of Human and Health Sciences, Swansea University, Singleton Park, Swansea, SA2 8PP UK; 7 Department of Experimental Psychology, Oxford Centre for Anxiety Disorders and Trauma, Paradise Square, Oxford, OX1 1TW UK; 8 Grenfell Emotional Health and Wellbeing service, Central and Northwest London (CNWL) NHS Trust, St Charles Hospital, Exmoor Street, London, UK; 9 Formerly at Institute of Psychotrauma, East London Foundation NHS Trust, 86 Old Montague Street, London, E1 8NN UK; 10 Department of Mathematics, Laver Building, University of Exeter, Exeter, EX4 4QRE UK; 11 Division of Nursing, Midwifery & Social Work, School of Health Sciences, Faculty of Biology, Medicine and Health, The University of Manchester, Room 6.322a, Jean McFarlane Building, Oxford Road, Manchester, M13 9PL UK; 12 Department of Psychology, University of Derby, Kedleston Road, Derby, DE22 1GB UK

**Keywords:** PTSD, Post-traumatic stress disorder, RCT, Randomised controlled trial, Guided self-help, Trauma focused, Intervention, Internet, Online, Protocol

## Abstract

**Background:**

There is good evidence that trauma-focused therapies for Post-Traumatic Stress Disorder are effective. However, they are not always feasible to deliver due a shortage of trained therapists and demands on the patient. An online trauma-focused Guided Self-Help (GSH) programme which could overcome these barriers has shown promise in a pilot study. This study will be the first to evaluate GSH against standard face-to-face therapy to assess its suitability for use in the NHS.

**Methods:**

The study is a large-scale multi-centre pragmatic randomised controlled non-inferiority trial, with assessors masked to treatment allocation. One hundred and ninety-two participants will be randomly allocated to receive either face-to-face trauma-focused cognitive behaviour therapy (TFCBT) or trauma-focused online guided self-help (GSH). The primary outcome will be the severity of symptoms of PTSD over the previous week as measured by the Clinician Administered PTSD Scale for DSM5 (CAPS-5) at 16 weeks post-randomisation. Secondary outcome measures include PTSD symptoms over the previous month as measured by the CAPS-5 at 52 weeks plus the Impact of Event Scale – revised (IES-R), Work and Social Adjustment Scale (WSAS), Patient Health Questionnaire-9 (PHQ-9), General Anxiety Disorder-7 (GAD-7), Alcohol Use Disorders Test (AUDIT-O), Multidimensional Scale for Perceived Social Support (MSPSS), short Post-Traumatic Cognitions Inventory (PTCI), Insomnia Severity Index (ISI) and General Self Efficacy Scale (GSES) measured at 16 and 52 weeks post-randomisation. Changes in health-related quality of life will be measured by the EQ-5D and the level of healthcare resource utilisation for health economic analysis will be determined by an amended version of the Client Socio-Demographic and Service Receipt Inventory European Version. The Client Satisfaction Questionnaire (CSQ) will be collected at 16 weeks post-randomisation to evaluate treatment satisfaction.

**Discussion:**

This study will be the first to compare online GSH with usual face-to-face therapy for PTSD. The strengths are that it will test a rigorously developed intervention in a real world setting to inform NHS commissioning. The potential challenges of delivering such a pragmatic study may include participant recruitment, retention and adherence, therapist retention, and fidelity of intervention delivery.

**Trial registration:**

ISRCTN13697710 registered on 20/12/2016.

## Background

Post-Traumatic Stress Disorder (PTSD) is a common mental disorder that may develop following exposure to exceptionally threatening or horrifying events. Characteristic symptoms include persistent intrusive recollections, avoidance of trauma-related stimuli, negative alterations in thoughts and mood and hyper-arousal [1, 2]. About 3% of the adult population suffer from current PTSD [3] and average symptom duration is normally prolonged in those who are untreated [4]. PTSD is associated with substantial co-morbidity [4–8] and significant economic burden [9, 10].

A number of psychological approaches have been developed to treat PTSD. Evidence suggests that the most effective approaches are trauma focused cognitive behavioural therapy (TFCBT), including trauma focused cognitive therapy, and eye movement desensitization and reprocessing therapy (EMDR) [11]. TFCBT typically involves some degree of structured exposure to and processing of traumatic memories and trauma reminders, alongside cognitive restructuring of dysfunctional beliefs about the meaning of the trauma. EMDR is a psychological therapy that involves exposure to unwanted and distressing memories whilst focusing on a bilateral stimulation. TFCBT and EMDR have become the treatments of choice for PTSD, recommended by clinical guidelines in the UK and internationally [12–14]. TFCBT protocols vary in the focus on exposure or cognitive interventions and differ slightly in the number of treatment sessions that are recommended. National Institute for Health and Care Excellence (NICE) recommend treatment of 8–12 sessions lasting 60–90 min for individuals who have experienced a single trauma, with a recommendation of more than 12 sessions in chronic, complex cases i.e. with multiple traumas.

Despite a growing consensus that trauma focused psychological therapies (TFPTs) represent the most effective way of treating PTSD, they are not always feasible to deliver. There remains a shortage of suitably qualified therapists to deliver these interventions and therefore, in many places, lengthy waiting times are common. If left untreated, PTSD is associated with functional and emotional impairment, reduced quality of life, a predisposition for the development of other psychiatric and physical illnesses, increased suicidal ideation, higher healthcare utilisation, and higher rates of alcohol abuse and dependence [15–21]. Current TFPT treatment requires a considerable commitment from the service user to attend weekly out-patient appointments on a regular basis over several months. Therapy can be difficult for some people to access, due to factors such as perceived stigma about attending mental health services, difficulty getting time off work, problems accessing or arranging suitable childcare and travel for people living in remote areas [22–24]. As a result of these and other factors, drop-out from TFPT interventions can be high [11].

Guided self-help (GSH) could offer a solution to these issues. GSH combines the use of self-help materials (e.g., a work-book, or a website), with regular guidance from a trained mental health professional and requires less therapist time than an equivalent therapist-administered treatment. There is good evidence of the efficacy of GSH in other mental health disorders [25]. In recognition of these findings NICE recommended that a randomised controlled trial (RCT) of GSH should be conducted to assess the efficacy and cost-effectiveness of guided self-help compared with trauma focused psychological interventions for mild and moderate PTSD [12]. If effective, GSH would offer a time-efficient treatment option with the potential to reduce waiting times and would be more accessible and less of a burden to participants. It is also likely that GSH would reduce intervention costs, and may lessen the burden of PTSD to the NHS and society.

Some of the authors (JB, CL, NR, NK) have systematically developed a novel, internet-based GSH programme for PTSD based on TFCBT. This was developed over a number of years following Medical Research Council (MRC) guidance for the development of a complex intervention [26] with significant input from PTSD sufferers and professional stakeholders. Intervention development work was completed between 2007 and 2010. A modelling phase included key stakeholders in focus groups and semi-structured interviews to inform the content, delivery and guidance of a GSH programme for PTSD. Data was analysed using qualitative methodology and used to inform the first prototype. The prototype was piloted twice with a total of 19 participants with PTSD and refined on the basis of qualitative and quantitative results. Quantitative results strongly supported the potential of the programme to effectively treat PTSD [27].

An interactive online version of the programme was produced through a Knowledge Transfer Partnership (KTP) between Health Care Learning Limited Ltd (HCL) and Cardiff University. The partnership combined HCL’s expertise in developing high quality internet-based programmes, with the academic team’s experience of developing and evaluating psychological interventions for PTSD. A pilot feasibility RCT of the intervention was completed between 2012 and 2014. Forty-two participants with mild to moderate PTSD after a single traumatic event were randomised to receive immediate GSH or delayed treatment [28].

Following GSH, PTSD sufferers’ symptoms improved by over 50% (completers only) and over 40% (intention to treat) with an average of 149 min of therapist input; effect sizes that compare favourably with those found for therapist-delivered TFPT. The treatment group had significantly lower levels of traumatic stress symptoms, depression, anxiety and functional impairment at post-treatment and one month follow-up, in comparison to the delayed treatment group, who improved to the same degree after receiving GSH for PTSD. Results of the development and pilot work indicated a strong rationale for conducting a larger scale RCT to determine whether GSH represents a treatment option that should be routinely used in the care of PTSD sufferers, as it is for depression and numerous anxiety disorders.

A Cochrane review of RCTs of online GSH for PTSD in comparison to face-to-face therapy, waitlist or usual care [29] found two previous studies [30, 31]. The first compared online GSH with 104 min of guidance to a waitlist in 42 adults with PTSD [30]. A large within group effect size was found in the GSH group from pre- to post-treatment for self-reported PTSD symptoms (Cohen’s d = 1.18) but a smaller between groups effect size was found post-treatment, due to symptom improvement in the control group. The second RCT compared online GSH to a delayed treatment minimal attention group in 62 PTSD sufferers [31]. A larger between group effect size of d = 1.25 was found. The interventions included in both studies were largely text-based and may not have been optimal in terms of usability. The online GSH intervention developed by this study team includes audio and video clips and is therefore more interactive which may explain the positive results seen in the pilot study.

The review demonstrated that there have been no comparative trials of online GSH and face-to-face therapy to date, precluding firm decisions being made on whether to deliver GSH for PTSD as an alternative to face-to-face therapy in the NHS. The proposed study will address this by generating high quality scientific evidence for an intervention already developed by us through state of the art methodology. Our aim is to test online GSH as a treatment for adults who have been exposed to a single traumatic event. The primary objective of the trial is to determine: Whether an online TFCBT based GSH programme is not inferior to individual TFCBT for patients with PTSD as judged by reduced symptoms of PTSD at 16 weeks post-randomisation, thus enabling decisions to be made about its suitability for use in the NHS. The secondary objectives of the trial are to determine:

1. Whether an online TFCBT based GSH programme is not inferior to individual TFCBT for patients with PTSD as judged by reduced symptoms of PTSD at 52 weeks post-randomisation.
2. Whether an online TFCBT based GSH programme is not inferior in effectiveness to individual TFCBT for patients with PTSD as judged by improved quality of life at 16 weeks and 52 weeks post-randomisation.
3. The impact of an online TFCBT based GSH programme on functioning, symptoms of depression, symptoms of anxiety, symptoms of PTSD, alcohol use, insomnia, perceived social support, self-efficacy and cognitions for people with PTSD at 16 weeks and 52 weeks post-randomisation.
4. Whether an online TFCBT based GSH programme is cost-effective relative to individual TFCBT for patients with PTSD at 16 weeks and 52 weeks post-randomisation.
5. The factors which may impact effectiveness and successful roll-out of online GSH for PTSD in the NHS if the GSH programme is shown to be effective.

## Methods/design

The design is that of a multi-centre pragmatic randomised controlled non-inferiority trial with assessors masked to treatment allocation. Individual randomisation will be used.

### Study setting

The trial will take place in NHS Improving Access to Psychological Therapy (IAPT) services based in primary care in England, and in NHS psychological treatment settings based in primary and secondary care in Scotland and Wales. The sites cover urban and rural, economically and non-economically deprived areas of the UK in Coventry, Warwickshire, Greater Manchester, London, South West Yorkshire, South Wales and Lothian.

### Eligibility criteria

Wide eligibility criteria will be used to ensure good external validity. Given the high rate of co-morbidity of PTSD and other conditions such as depression and substance misuse, individuals with co-morbidity will be included if they satisfy the other inclusion/exclusion criteria and PTSD is considered the primary diagnosis. This is consistent with NICE guidance for the treatment of PTSD [12] and will result in a pragmatic trial. See Table 1 for Inclusion and Exclusion Criteria.

**Table 1:** RAPID Inclusion and Exclusion Criteria

*Inclusion Criteria*
Participants must be:
1) Aged 18 or over
2) Screen positive for PTSD to a single traumatic event on the Trauma Screening Questionnaire (TSQ)
3) Have regular access to the internet in order to complete the steps and homework required by the GSH programme
4) Be willing and able to give informed consent to take part
5) After a 2 week monitoring period, continue to meet CAPS-5 criteria for mild to moderate PTSD (score less than 50)
6) Have PTSD as their primary diagnosis.
*Exclusion Criteria*
A person is not eligible to enter the trial if any of the following apply (all of these are measured via self-report):
1) Inability to read and write fluently in English
2) Previous completion of a course of TFPT for PTSD
3) Currently engaged in a psychological therapy
4) Change in psychotropic medication in the last 4 weeks
5) Psychosis
6) Substance dependence
7) Active suicide risk

With reference to inclusion criterion 2, previous work with more complex and severe forms of PTSD, e.g. following prolonged and repeated trauma suggests that it often requires increased therapist time and is therefore less likely to be effectively treated by GSH [12]. In terms of criterion 5, symptoms can remit when monitored, hence the 2 week self-monitoring period in which participants will be asked to complete a simple daily diary of symptoms at home [32, 33]. Participants cannot engage in concurrent psychological therapy whilst in the trial. They can continue taking psychotropic medication but are not eligible if they have had a change in medication 4 weeks prior to being assessed.

### Interventions

The trial will compare online GSH against individual face-to-face TFCBT, which is current standard care in the sites taking part in the trial.

#### TFCBT for PTSD

TFCBT for PTSD is one of the standard treatments adopted by IAPT in England and has been shown to be effective in RCTs in England [11, 33–36] and Northern Ireland [37]. There are a number of evidence based TFCBTs and, given the pragmatic nature of the trial, it will be permissible to use any one of them with individuals allocated to the TFCBT arm. An example of an evidence-based TFCBT is cognitive therapy for PTSD (CT-PTSD). CT-PTSD involves identifying the relevant appraisals, memory characteristics, triggers, and behavioural and cognitive strategies that maintain PTSD symptoms. CT-PTSD addresses these symptoms by: 1) Modifying excessively negative appraisals of the trauma and/ or its sequelae, 2) Reducing re-experiencing by elaboration of the trauma memories and discrimination of triggers, 3) Dropping dysfunctional behaviours and cognitive strategies, particularly those related to avoidance of triggers for intrusive symptoms. These are strategies that have the immediate aim of reducing one’s sense of current threat but have the long-term effect of maintaining the disorder, and are common in PTSD.

In this study, TFCBT will be delivered by experienced psychological therapists currently working in the IAPT or psychological services. They come from a variety of backgrounds, including mental health nurses, clinical psychologists and counsellors, and have varying levels of experience in working therapeutically with people with PTSD. All therapists will attend at least 1 one-day workshop on one of the TFCBT programmes. In addition, all study therapists are required to attend at least one and a half days training in CT-PTSD delivered by the group who developed CT-PTSD and to be assessed as being at least satisfactorily competent in the delivery of TFCBT by one of the two supervising authors who are experienced in TFCBT (NK, NR). The therapists will be given a treatment manual and will receive supervision once per month from one of two authors (NK, NR).

TFCBT will be delivered face-to-face to individual participants over the course of up to 12 sessions, each lasting 60–90 min. Appointments will take place at the service base. In-session treatment is augmented by daily homework assignments which participants are required to complete between sessions. They will be asked to complete the Impact of Events Scale-Revised (IES-R) at each session to measure and monitor their PTSD symptoms.

#### Spring: An internet based guided self-help Programme for PTSD

Spring is an online GSH programme based on TFCBT. It uses the same principles as TFCBT but aims to reduce contact time with the therapist by providing some of the therapy content and activities in an accessible self-help format. Potential advantages include that the service user can engage in the intervention at a time and place convenient to them which may reduce drop out, and it may also reduce waiting list times as it should reduce the input time required for therapist.

The expectation is that Spring will be delivered by the same therapists delivering the TFCBT. All the therapists will receive a half day’s training from two of the authors who were involved in developing the intervention and delivering it in the pilot study (NK, NR). Therapists also receive ongoing supervision of one or more training cases until they are regarded as competent. The therapists will work from a manual and, for the duration of the trial, will receive supervision once per month from NK or NR.

Spring is an 8 week online program comprising eight steps (see Table 2. Spring PTSD Steps).

**Table 2:** Spring PTSD Steps

Step 1: Learning About My PTSD – Psychoeducation about PTSD illustrated by four actors describing their experience of PTSD to different types of traumatic event.
Step 2: Grounding Myself - Explanation of grounding and its uses along with descriptions and demonstrations of grounding exercises.
Step 3: Managing My Anxiety – Education about relaxation techniques with learning through videos of a controlled breathing technique, deep muscular relaxation and relaxation through imagery.
Step 4: Reclaiming My Life – Behavioural re-activation to help individuals return to previously undertaken/new activities.
Step 5: Coming to Terms with My Trauma – Provides rationale for imaginal exposure, narratives of the four video characters. The therapist helps the participant to begin writing a narrative, which they complete remotely and read every day for at least 30 min.
Step 6: Changing My Thoughts – Cognitive techniques to address PTSD symptoms.
Step 7: Overcoming My Avoidance – Graded real life exposure work.
Step 8: Keeping Myself Well – This session reinforces what has been learnt during the programme, provides relapse prevention measures and guidance on what to do if symptoms return.

**Fig. 1:**
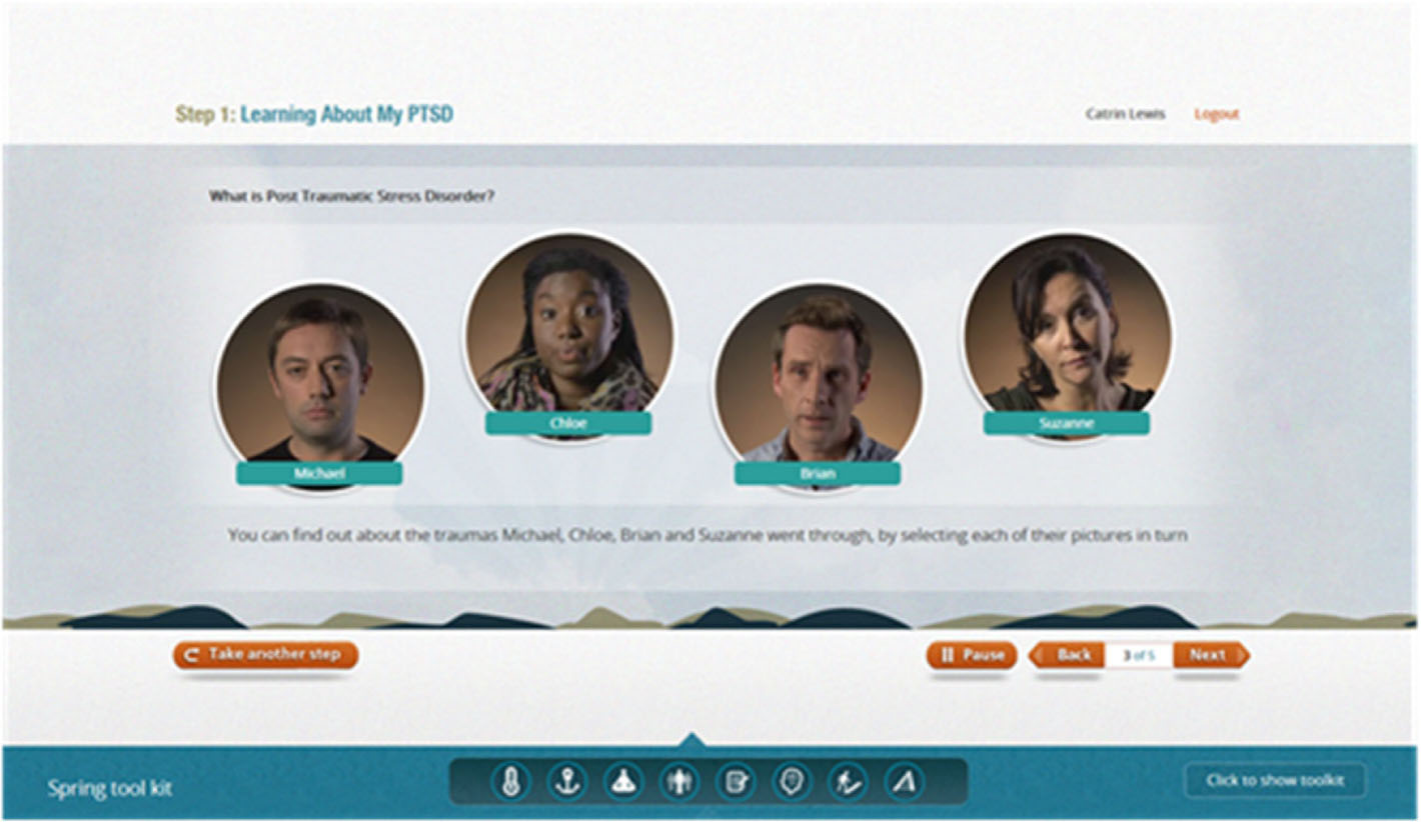
Spring Screenshot. A screenshot, taken from Step 1, showing the actors whose PTSD case histories are followed throughout the intervention. The Toolkit can be seen at the bottom of the webpage

The therapist initially meets with the participant at the service base for an hour to develop a rapport, learn about the participant’s trauma and describe the programme, which the participant then completes online in their own time. There are four subsequent fortnightly meetings of 30 min, normally undertaken face-to-face, but potentially deliverable via the internet or telephone, according to participant preference. The participant will also receive four brief telephone calls or email contact between sessions to discuss progress, identify any problems that have arisen and sometimes set new goals. The modules are accompanied by homework. At each session, the therapist reviews progress by logging into a clinician dashboard, and guides the participant through the programme. The aim of the guidance is to offer continued support, monitoring, motivation and problem solving. The eight online steps are usually completed in turn with some later steps relying on mastery of techniques taught in earlier steps. Each step provides psycho-education and the rationale for specific components of treatment. Each step activates a tool that becomes live in the Toolkit area of the website and aims to reduce traumatic stress symptoms. Everything entered into the Toolkit becomes visible (with the participant’s knowledge) to the therapist via the dashboard to facilitate input. The programme can be accessed online via a web browser or through an App. Participants will be able to use their PCs, tablets or smart phones to engage with the programme. A screenshot taken from Step 1 can be seen in Fig. 1: Spring Screenshot. Participants will be asked to complete the IES-R at each face-to-face session to measure and monitor their PTSD symptoms.

### Participant withdrawal

A participant may withdraw or be withdrawn from either intervention for the following reasons: 1) Withdrawal of consent to participate in the intervention by the participant, 2) Any alteration in the participant’s condition which justifies the discontinuation of the intervention in the therapist’s/Principal Investigator’s opinion. For example, developing a condition which would exclude them from the study based on the eligibility criteria.

In the case of suicidal ideation, the therapist will assess the participant to determine whether or not they can continue or need to be referred elsewhere. In the case that a participant scores 50+ on the CAPS-5 at follow-up, they will also need to be assessed by the clinical team and may be offered additional treatment. Participants who solely withdraw/are withdrawn from the intervention will continue in follow-up unless they withdraw their consent for this.

Participants can chose to withdraw from the trial intervention, withdraw from follow-up, withdraw from both aspects, or withdraw from both aspects and ask that previously collected data not be used.

### Fidelity

To ensure the interventions are delivered as intended and according to the manuals, each therapist will aim to audio record at least one session with every participant using a digital recorder (however, this may not be pragmatic for GSH if the sessions are solely conducted on the telephone or by email). The audio recordings will be rated using a general and an intervention specific fidelity checklist by one of two independent raters, who have received training in the intervention. We will record the number of sessions attended or missed for each participant, and the time spent on the GSH programme to measure adherence.

### Outcomes

The primary outcome will be the severity of symptoms of PTSD over the previous week as measured by the Clinician Administered PTSD Scale for DSM5 (CAPS-5) [38] at 16 weeks post-randomisation. The CAPS-5 is a 30 item structured interview for assessing PTSD diagnostic status and symptom severity. It is the gold standard in PTSD assessment and can be used to make a current (past month) or lifetime diagnosis of PTSD or to assess symptoms over the past week. Items correspond to the DSM5 criteria for PTSD. Previous versions of the CAPS have excellent reliability and convergent and discriminant validity, diagnostic utility, and sensitivity to clinical change [39]. Sixteen weeks was chosen as a post-intervention measurement.

Secondary outcomes include the severity of PTSD symptoms at 52 weeks post-randomisation to determine whether any effects of the intervention are sustained, and will be measured using the CAPS-5 1 month version. Other secondary outcomes to be measured at both 16 weeks (to determine the effect of the intervention) and 52 weeks post-randomisation (to determine sustained effects) will include symptoms known to be commonly associated with PTSD along with functional status. To assess these, we will use self-report measures that are routinely collected by IAPT services: 1) Traumatic stress as measured by the Impact of Event Scale – revised [40], 2) Quality of Life/functional impairment as measured by the Work and Social Adjustment Scale [41], 3) Depression measured by the Patient Health Questionnaire-9 [42], 4) Anxiety measured by General Anxiety Disorder-7 [43] and 5) Alcohol use measured by AUDIT-O [44].

Additional, non-IAPT measures will be administered at 16 and 52 weeks post-randomisation. Perceived social support will be measured using the Multidimensional Scale for Perceived Social Support [45], and the level of healthcare resource utilisation for health economic analysis will be determined by an amended version of the Client Socio-Demographic and Service Receipt Inventory European Version [46]. Changes in health related quality of life will be measured by the EQ-5D [47] and changes in sleep measured by the Insomnia Severity Index [48]. A short version of the Post-Traumatic Cognitions Inventory [49] and the General Self Efficacy Scale [50] will be collected to determine effects on cognitions and self-efficacy respectively. The Client Satisfaction Questionnaire [51] will be collected at 16 weeks post-randomisation only, to evaluate treatment satisfaction.

The IES-R will be collected at each therapy contact to provide clinical feedback and also to facilitate imputation for missing data, if required. How frequently it is collected will depend on the frequency of therapist contact, but is likely to be weekly in the TFCBT arm and fortnightly in the GSH arm.

#### Process evaluation

A process evaluation conducted alongside the main trial will explore contextual factors and mechanisms of change that may impact on effectiveness and successful rollout of the intervention post-trial. Specifically, we will examine the contextual factors surrounding intervention delivery, which will include assessment of recruitment, retention, fidelity and adherence. The process evaluation will be developed according to the MRC guidance [52] and will make use of both quantitative data (including fidelity measurement, retention, adherence rates, time spent on different steps of the GSH programme) and qualitative data from interviews with therapists and participants pre- and post-intervention. Detailed information regarding whether the intervention was delivered as intended (fidelity) and the quantity of the intervention implemented (dose) will allow us to test the theorised mechanism of effect of GSH for PTSD (a combination of: psycho-education about PTSD; imaginal and in-vivo exposure work to achieve habituation to distressing images and avoided situations; cognitive work to identify and modify negative/distorted cognitions; and stress management skills to cope with anxiety and other symptoms) and whether certain factors appear to be more important than others.

### Sample size

As the study aims to demonstrate non-inferiority of GSH for PTSD compared to TFCBT, the power calculation considers the non-inferiority margin as opposed to the effect size. The non-inferiority margin will be 5 points on the 80 point CAPS-5 scale. A recent meta-analysis [11] indicates that the standardised mean difference between TFCBT and waitlist/usual care for the treatment of PTSD is − 1.62. This corresponds to 16.6 points on the CAPS-5. This means that if we demonstrate non-inferiority to within 5 points of the gold standard, we will also demonstrate superiority over wait list/usual care in line with International Conference on Harmonisation Harmonised Tripartite Guideline (Statistical Principles for Clinical Trials) E9 (ICH E9) guidance for non-inferiority studies [53, 54].

Pilot work indicates an intraclass correlation coefficient of 5.6% at the therapist level at 10 weeks. At 22 weeks, however, there was no observable clustering of CAPS-5 scores amongst therapists. Given our primary outcome (CAPS-5) is measured at 16 weeks we have allowed for 1% clustering and recalculated the sample size. We have allowed for 20% attrition. On the basis of the anticipated average therapist cluster size being four, the design effect is 1.03, requiring a 3% inflation of the sample size. This results in a final sample size of 192 (inflated from 186) which provides 90% power (nQuery v7.0).

For the qualitative elements of the study, the sample size will be guided by preliminary analysis and constant comparison (comparing and contrasting themes from other interviews) during each data collection phase, until the research team is satisfied that there is data saturation and no new themes which are important to the research question arise [55]. However, it is helpful to have a guide to sample size for study planning. Based on previous research [56] we propose that interviews will be conducted with 10–20 participants and 8 therapists purposefully sampled from the different geographical sites.

### Recruitment

**Fig. 2:**
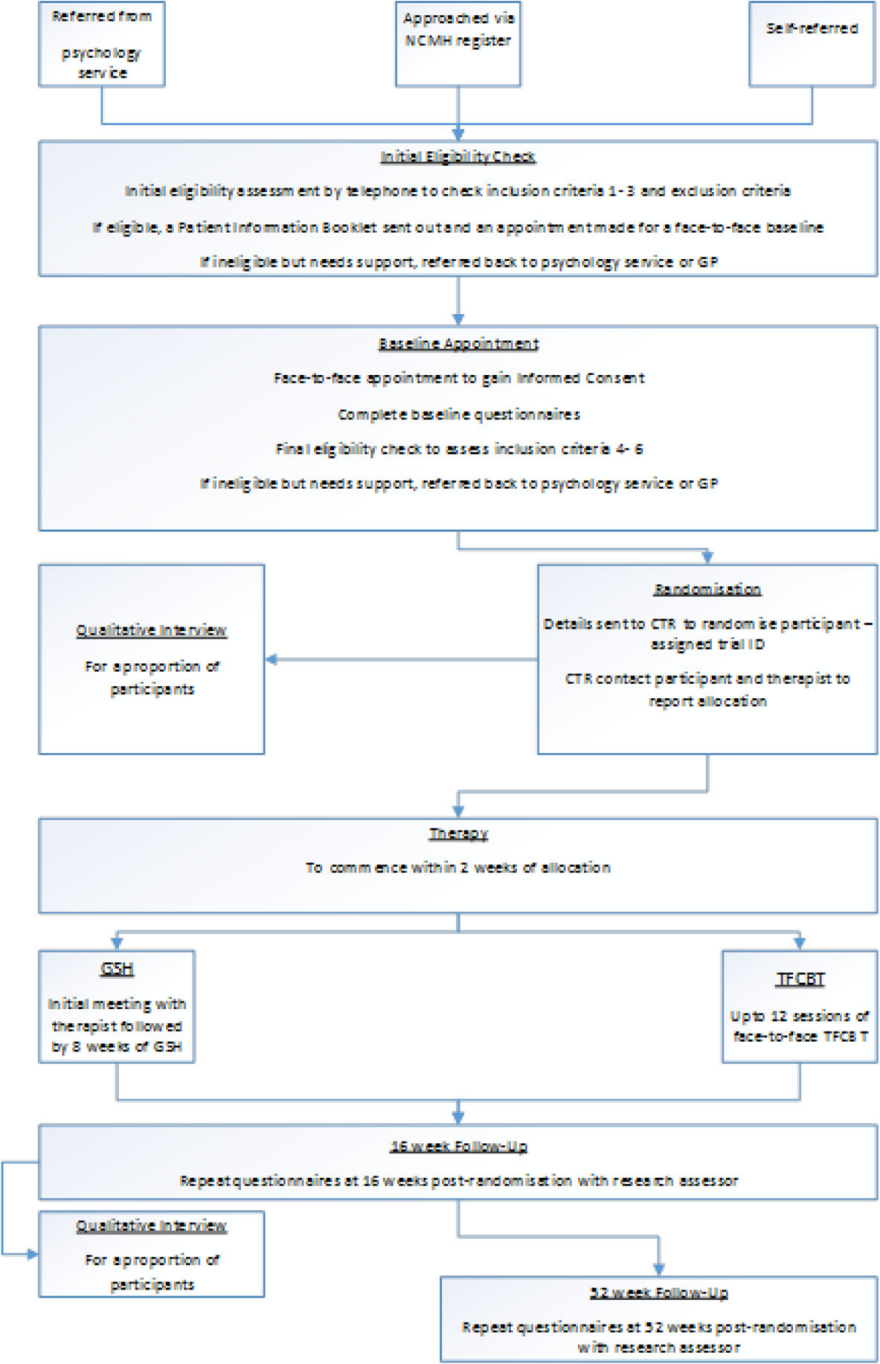
Participant Timeline. Participant activity throughout the trial

Please refer to Fig. 2: Participant Timeline, for participant activity throughout the study. Prospective participants will be identified through Primary Care Mental Health Services (PCMHS) in South Wales, through IAPT services in England and psychological treatment services in Scotland. Three centres in Cardiff, Manchester and Musselburgh will initially oversee recruitment at up to 3 sites each and one site in London. More sites may be added if necessary. Primary care and other relevant workers in these services will be educated about the study and the eligibility criteria, and asked to identify and refer patients who may be experiencing PTSD to a single traumatic event and meet two of the other eligibility criteria (aged 18+ with regular access to the internet). They will be provided with a Summary Information sheet of the study, which they can discuss with the patient. With the patient’s consent, they will pass their contact details to the researchers by secure fax or a telephone call. The researcher will then telephone the patient to confirm they are over 18 years old with regular internet access, screen positive for PTSD on the Trauma Screening Questionnaire (TSQ) and do not appear to meet any of the exclusion criteria. If the patient is probably eligible, the researcher will send them a copy of the Participant Information Booklet to read before the appointment. If the patient is found to be ineligible, they will be referred back to the referring service. The referring services will be contacted regularly and will be provided with trial branded merchandise such as pens to remind them about the study.

If necessary, to obtain the number of participants required, the Cardiff centre may also recruit participants through the National Centre for Mental Health (NCMH) cohort of participant volunteers. The cohort of over 9500 participants with lived experience of a mental illness currently includes 890 individuals with a diagnosis of PTSD, the majority of whom live in South Wales. A member of the NCMH team would contact potentially eligible individuals who have consented to being contacted about future research, to screen for eligibility and invite participation if eligible.

In addition to the methods described above, individuals referred to the local tertiary traumatic stress services will be screened by service clinicians and details of potentially eligible patients passed to the research team as described above. Information about the study will be communicated across all primary and secondary care services (including counselling services) in the recruitment areas and University Student Support Services (these were a source of recruitment in the pilot study). A limited number of leaflets will be available at key NHS services such as IAPT services, GP surgeries, Accident and Emergency services and outpatient clinics. We will publicise the trial publicly in conjunction with the NCMH communications team. This will include targeted press releases to local media with the offer of an interview with one of the study team, news items and advertisements on the NCMH website (www.ncmh.info), and a social media campaign to raise awareness of the study. We will seek to explore opportunities to recruit through the Criminal Justice System by linking with Victim Support, Sexual Assault Referral Centres (SARCs) and HM Court Services.

A screening log of all ineligible and eligible but not consented/not approached will be kept at each site so that any biases from differential recruitment will be detected.

### Allocation and blinding

Where possible, baseline measures will be collected electronically using an online database. Following completion of the baseline assessment, the database will inform the assessor that the participant is eligible for randomisation and the assessor will click the ‘Randomise Participant’ button. The database will allocate the participant to either GSH or TFCBT using pre-programmed software. This information will not be available to the assessor, who is blind to allocation, instead the database will email the trial manager who will inform the appropriate therapist. Individual randomisation will be conducted using an online minimisation algorithm generated by the trial statistician. The ratio will be 1:1. Minimisation will ensure balance between trial arms for gender, but will retain a random element and will be stratified by research centre.

It is not possible to blind the therapists or the participants given the complex interventions under investigation. However, the outcome assessors will be blind to treatment allocation and the therapists and participants will be asked not to discuss their allocation with the assessor. This will be stressed at the start of the interview and will minimise any potential bias the assessor may have when conducting the outcome assessments. To measure the success or otherwise of allocation concealment, the assessors will be asked to guess the participant’s allocation before and after each assessment. This will be recorded and compared to chance at the end of the study.

### Data Collection Methods & Data Management

All assessments will be conducted by research assessors who are either employed as Research Assistants by the centres involved, or are Clinical Studies Officers from the local Clinical Research Network. They will all receive training in rating the outcome measures. For the CAPS-5, this includes at least 1 day full training followed by rating a recording of an interview between an actor and an experienced interviewer. Assessors could be signed off if they score within 10% of the total score agreed by two of the authors (JB, CL), score differently on a maximum of four items and do not score more than one point different on any individual item. In order to ensure accurate ratings, assessors will receive video CAPS-5 interviews every 6 months to rate and return to JB and CL who will provide feedback.

Once they receive a referral, assessors will contact the potential participant by telephone to conduct a screening questionnaire made up of the TSQ and simple questions to assess inclusion criteria 1–3 and all of the exclusion criteria. Eligible participants will be sent a Participant Information Booklet and a daily diary and will be asked to monitor their symptoms for 2 weeks, as previous studies have found that this results in significant reduction in symptoms for some PTSD sufferers [33].

After the 2 week monitoring period, those still interested in taking part will be asked to attend a face-to-face appointment. Assessors, who have been trained in Good Clinical Practice (GCP) and receiving informed consent, will begin the consent process with the participant by reading through the Patient Information Booklet together and giving them to time to ask questions about the study. The assessor will then ask the participant to complete the triplicate study Consent Form if they are happy to do so. One copy will be given to the participant to keep. The assessor will then conduct the baseline assessment, which will assess inclusion criteria 4–6 and re-check criteria 2 (single event) and exclusion criteria 7. They will collect demographic data and the outcome measures previously outlined. Follow-up assessments will occur, as close as possible, to 16 weeks and 52 weeks post-randomisation. This will involve re-administration of the outcome measures and may be conducted face-to-face or on the telephone.

To improve data quality and completeness we have developed a study specific online database which can be accessed by all of the assessors. During the assessments, participants are encouraged to complete the self-report questionnaires directly onto the computer and assessors also complete the remaining parts of the interview online. The database flags up any missing variables, or entries outside the permissible range, thus ensuring complete and accurate data. Access to the database will be restricted to named study personnel only via a secure login and password. The system will be roles-based with restricted read/write/edit permissions. Any changes made to the data will be stored in the audit log within the system’s database with a full history of changes being recorded. The system will be accessible via any online PC, tablet or mobile device. Data will be stored securely on a secure server. In case of lack of access to an internet connection, we also have back-up paper case report forms which are entered onto the database as soon as possible. When paper copies are used, a copy will be sent to the trial co-ordinating centre and the data entered will be verified.

Qualitative interviews will be conducted with a sample of participants once they have consented to the trial but ideally prior to starting the intervention, and then again after the intervention. A sample of therapists will also be interviewed prior to commencing delivery of the interventions and again on completion. All interviews will be digitally audio-recorded then transcribed by a member of the study team.

To aid retention, participants will be offered a £10 shopping voucher on completion of the 16 week and 52 week follow-up assessments as a token of appreciation for their participation in the study. Where a participant wishes to withdraw from the intervention, their participation in the follow-up assessments will be encouraged. Unless a participant has withdrawn consent for follow-up, repeated attempts using different approaches will be made to contact participants who cannot be easily contacted. In a step-wise manner, this will involve checking contact details with their study therapist, calling the individual on all contact numbers provided on various days of the week and at different times, sending emails and a letter to the addresses provided. If contact can still not be made, the individual’s GP will be contacted to check contact details are correct. If these attempts do not result in contact being made within 1 month of loss of contact or the planned follow-up, a letter will be sent asking the participant to re-establish contact if they are able to and advising that they will be contacted again at the next follow-up point unless they advise otherwise. For any participant reluctant to complete the full outcome assessment at follow-up we will attempt to gain the CAPS-5 information as a minimum dataset. As much information as possible will be collected from protocol non-adherers, with a minimum of the primary and secondary outcome measures and reasons for non-adherence.

### Statistical methods

The primary analysis will be performed using analysis of covariance, predicting follow-up CAPS-5 score controlling for baseline CAPS-5 score and important patient characteristics (including all minimisation variables). This will be a complete case intention to treat analysis. Checks will be made to ensure there is no appreciable clustering of outcomes within therapists, but if such clustering exists the primary analysis will be hierarchical. The results will be summarised using point estimates, 95% confidence intervals and *p*-values. Since this is a non-inferiority design, we will be checking whether the confidence interval for the difference between arms lies entirely within the 5 point non-inferiority margin. Participants with missing CAPS-5 score at follow-up will have a CAPS-5 score estimated from available IES-R scores (this will involve building a prediction model using information from participants with both IES-R and CAPS-5 scores). We will explore differences in treatment effects by gender in a sub-group analysis by including an interaction term between treatment arm and gender.

A sensitivity analysis will use multiple imputation to account for missing data if the number of cases lost due to incomplete information exceeds 10%. Secondary outcomes include: CAPS-5 at 52 weeks, IES-R, WSAS, PHQ-9, GAD-7, AUDIT-O, MSPSS, EQ-5D, ISI, GSES, PTCI, adapted CSSRI-EU and CSQ-8. These are all continuous measures and will be analysed similarly to the primary outcome. Transformations will be explored to improve model fit if distributional assumptions are not satisfied. This will be assessed by visual inspection and formal fit statistics compared to decide on the transformation chosen.

IES-R scores over time will be explored using a hierarchical modelling (including clustering by therapist if this is identified in the primary analysis) and an appropriate covariance structure allowing for IES-R scores within an individual to be correlated over time. Covariance structures to be explored include autoregressive terms (AR), moving average (MA), and combined terms (ARMA). This will facilitate the fitting of IES-R trajectories over time (since randomisation) interacted with intervention arm, whilst also controlling for the same covariates as the primary analysis. A sensitivity analysis will use multiple imputation to account for missing data if the number of cases lost due to incomplete information exceeds 10%. A further sensitivity analysis will account for patient adherence to the protocol using complier adjusted causal effect (CACE) analysis [57]. All analyses will be performed in the R programming language and environment or SPSS [58, 59].

Qualitative data will be analysed using framework analysis [60]. This is a systematic five-stage method, which is increasingly being used in health care research [61]. It will allow us to compare themes across time point, treatment centre, and interviewee category (i.e. patient and therapist). We will identify contradictory data, as points of contrast as well as similarities will be important in order to understand uptake of the GSH tool. The method is well defined and allows for greater transparency. Vital measures will be put into place to ensure validity and reliability. More than one person will be involved in the analysis and double coding will be carried out until consensus is reached. The framework analytic approach has been selected as it is a recognised transparent analytic approach. This qualitative component has been designed using the principles of the Critical Appraisal Skills Programme qualitative checklist, to ensure the quality of qualitative research [62].

### Health economics

An economic evaluation will be conducted from the perspective of the UK NHS and personal, social services. To determine the cost-effectiveness of GSH versus CBT for PTSD, and the extent to which it can be regarded as representing value for money, two analyses will be undertaken – one will assess the relative cost-effectiveness by estimating the incremental costs of achieving changes in natural units of outcome that commissioners, health care professionals, public health decision makers and service users find relevant (e.g. incremental cost of achieving a percentage improvement in PTSD symptoms as measured by CAPS5). The second analysis will use the EQ-5D utilities for a cost utility analysis estimating the incremental costs per quality adjusted life year (QALY) gained. The QALY gains will also be used in a net–benefit analysis based on accepted NICE ‘value for money’ thresholds.

The contributions associated with the GSH in relation to staff time and costs associated with training of therapists, along with materials and equipment used in the process associated with GSH development and implementation, will be collected during the trial by interviews with relevant finance staff, logged in physical units and translated into costs using published unit costs [e.g. [63, 64]. Resource utilisation of services prior to the GSH implementation, as a result of the intervention, and at follow-up relative to the control group, will be measured using the CSSRI-EU.

As the follow-up is at 52 weeks, no costs and outcomes will be subjected to discounting. Uncertainty around the cost and effectiveness estimates will be investigated by: probabilistic sensitivity analysis, using the incremental cost per QALY as the metric for this assessment measured against the NICE range of ‘willingness to pay’ thresholds between £20 k and £30 k per incremental QALY gained represented by cost-effectiveness acceptability curves [65]. a series of one-way sensitivity analyses to assess the impact of parameter variation on baseline estimates of the range of incremental cost-effectiveness ratios; and a set of alternative scenarios will be constructed, based on the findings from relevant studies of CBT for PTSD [33, 35–37] to compare the relative cost-effectiveness of GSH against different durations of CBT and supportive care .

In addition to a trial-based analysis, longer-term cost-effectiveness will be assessed using decision analytic modelling methods. The derived model, based on a review of published models at the time of the analysis, will use parameter estimates derived from the trial and information from literature sources relating to long-term effects of PTSD, alongside other sources, to arrive at meaningful long-term estimates of cost-effectiveness and budget impact.

### Trial monitoring

The trial will be monitored by a Trial Steering Committee (TSC) made up of independent members including lay members who will meet annually. We will also form a Data Monitoring Committee again made up of members independent of the trial. There are currently no planned interim analyses or stopping criteria.

All Serious Adverse Events (SAEs) must be reported immediately (and within 24 h of knowledge of the event) to the co-ordinating centre’s safety team. In addition to the usual SAE categories, for the purposes of this trial severe self-harm and harm to others must be reported. Therapists will be asked to notify the study team directly should they be concerned at any time that a participant has caused, or is likely to cause, significant harm to themselves. The therapist should also inform the participant’s GP. Therapists will be asked to inform the appropriate authorities directly should they become concerned at any time that a participant has, or is likely to cause significant harm to others. Assessors will be asked to complete a brief risk assessment if the participant discloses suicidal ideation and should discuss with a member of the clinical team what action to take.

Screening, recruitment, withdrawal and SAEs will be centrally monitored on a weekly basis. Database validations will be checked every 2 months and all consent forms received at the site will be checked. Following any amendments to the protocol or associated documents, the sites will be sent new versions and asked to confirm receipt. Their confirmation will be recorded on the relevant log. On site monitoring will occur only if a visit is triggered, for example, repeated protocol or GCP breaches.

### Data protection and indemnity

All personnel involved in the trial will act to preserve participant confidentiality and will not disclose or reproduce any information by which participants could be identified, except where specific consent is obtained. Data will be stored in a secure manner and will be registered in accordance with the Data Protection Act 1998. The data custodian and the translational sample custodian for this trial is Cardiff University. The Trial Manager, the Database Programmer and Statistician will have access to the final dataset.

Where participants are recruited at NHS sites the NHS indemnity scheme/NHS professional indemnity will apply with respect to claims arising from harm to participants at site management organisations.

### Dissemination

Trial findings will be disseminated widely using a variety of tailored methods targeting specific audiences. A summary report of trial results written in lay language will be sent to study participants and other key stakeholders. The report will also be displayed and available at venues used for recruitment. We will hold informal patient-centred meetings at each trial site, to present the results orally and allow time for questions and clarification. We will also hold an open conference at each centre in the final month of the project. If the outcome is positive, the conferences will include free training in the GSH programme for NHS staff coupled with free access to the programme for a certain number of therapeutic encounters. We will send reports of trial results to NHS commissioners, outlining the cost-saving potential of GSH if applicable and the scope for improving routine clinical practice for PTSD. We will disseminate the findings publicly through news items on the NCMH website (www.ncmh.info), which attracts an average of 2250 unique visitors each month, and an article in the widely circulated NCMH newsletter.

We will publicise the trial results through social media and publish posts related to trial progress and results on the NCMH blog-site. This site features posts that have attracted up to 10,000 hits. We have experience of successfully engaging local and national media and will work with the NCMH communications team to formulate strategies for press releases and the dissemination of findings through newspaper articles and radio features. We will work with knowledge brokers, such as the Science Media Centre, to maximise coverage. Study outcomes will be presented to the academic community at national and international conferences by means of oral presentation, poster presentation, and interactive workshops. We will target conferences likely to be attended by large numbers of therapists and managers working in IAPT and other primary and secondary care NHS psychological treatment services. We will also disseminate to the third sector and other services likely to deal with individuals with PTSD who could potentially benefit from treatment (e.g. SARCs, Victim Support). We aim to publish the quantitative, qualitative and health economic results in high impact, open-access, peer reviewed journals. A complete account of the research will also be published in the NIHR HTA Journal.

All the dissemination and promotion activities will be supported by project specific webpages on the NCMH website. The webpages will include descriptions of the project, its progress and achievements in plain and scientific language, press releases and announcements of and registration for conferences and training events. External evaluation of dissemination plans, including the identification of successful implementation strategies and barriers to implementation among end users (e.g. PTSD sufferers, health service planners and managers, clinicians, clinical professional bodies, etc.) will be undertaken by the TSC.

## Discussion

### Strengths of the study

This will be the first study to compare GSH for PTSD to a face-to-face therapy and will provide valuable information on which the NHS can make decisions about its suitability for use. The GSH intervention under investigation has been rigorously designed and tested and shown to be effective in pilot work. The study team are highly experienced in PTSD research and include the designers of TFCBT and GSH interventions, and the trial is being co-ordinated by an experienced Clinical Trials Unit (Centre for Trials Research, Cardiff University) with thorough Standard Operating Procedures. They will oversee rigorous data collection through the use of an electronic database designed to minimise missing or inaccurate data. The trial is multi-centre across England, Scotland and Wales and will be trialled in the services it would be delivered in if adopted by the NHS, thus ensuring its applicability to real life. The therapists have received thorough training and supervision in both TFCBT and GSH, and adherence to the protocol manuals will be checked by fidelity assessors. In addition, the assessors will use the CAPS-5, the ‘gold standard’, to measure PTSD symptoms and have all received training and feedback in using this assessment. Finally, a patient representative has been involved in all stages of the design of the study, has reviewed the GSH intervention and participant materials and sits on the Trial Management Committee.

### Challenges

This will be a pragmatic study conducted in a real-world NHS setting. Whilst this offers advantages in terms of applicability, it can also present unique challenges which can be broadly classified into three categories. The first is participant recruitment, retention and adherence. We know that people with PTSD can experience high anxiety and may at times find it difficult to leave the house, use public transport or travel to new places. This could mean a reluctance to take part or missed appointments, making it difficult to deliver the interventions as intended. This is particularly the case for the South Wales participants from two health boards who will be travelling considerable distances to Cardiff for treatment. This will be addressed with participants before they are randomised, to ensure ability to fully commit to the study.

Secondly, therapist retention may be a challenge. All therapists delivering the interventions are NHS employees with high caseloads, and taking part in the research is an additional responsibility. As such, there may be times when it is difficult for them to attend the study specific supervision sessions or site initiation visits, and the research team will need to be flexible in terms of delivering these. If a therapist does need to drop out of the study due to other work commitments or for personal reasons, it will not be a quick process to replace them, given the training requirements. Therefore, we plan to train several therapists at each site to minimise the risks of a site needing to close to recruitment. This also applies to assessors: several of our assessors are employed by the CRN and therefore we are subject to them being able to provide ongoing support.

Thirdly, there may be issues around the fidelity of the delivery. The therapists in the study have prior training in additional therapies and may bring elements of these into their sessions with participants. Conversely, they may not utilise all the tools available in TFCBT or GSH. To assess fidelity to the intervention, therapists have been asked to audio record at least one session from every participant. Whilst this would ideally have been every session, the limited availability of costly encrypted audio recorders meant this is not feasible.

All of these potential challenges will be thoroughly evaluated in the process evaluation.

## Conclusion

In summary, previous work has shown that trauma focused online GSH has the potential to be an effective treatment for PTSD. No study to date has compared GSH to face-to-face therapy. This study will be the first to compare the two, thus allowing the NHS and other health services and professionals to make decisions about the suitability of GSH as a treatment option. The study’s strengths are that it is led by a highly experienced team of PTSD researchers in conjunction with a trials unit and is conducted in a real world setting, thus ensuring it is applicable to the intended recipients. The challenges will be participant recruitment, retention and adherence, therapist retention and fidelity of intervention delivery.

## Abbreviations

AR: Autoregressive
AUDIT-O: Alcohol use disorders identification test outcomes
CACE: Complier adjusted causal effect
CAPS-5: Clinician administered PTSD scale for DSM5
CSQ-8: Client satisfaction questionnaire
CSSRI-EU: Client socio-demographic and service receipt inventory
DSM5: The diagnostic and statistical manual of mental disorders, fifth edition
EMDR: Eye movement desensitisation and reprocessing
EQ-5D-5 L: EuroQol-5 dimensions-5 levels
GAD-7: Generalised anxiety disorder
GCP: Good clinical practice
GSES: General self efficacy scale
GSH: Guided self-help
HCL: Health care learning
IAPT: Improving access to psychological therapy
ICHE9: International Conference on Harmonisation Harmonised Tripartite Guideline (Statistical Principles for Clinical Trials) E9
IES-R: Impact of event scale revised
ISI: Insomnia severity index
MA: Moving average
MRC: Medical Research Council
MSPSS: Multidimensional scale for perceived social support
NCMH: National Centre for Mental Health
NHS: National Health Service
NICE: National Institute for Health and Care Excellence
NIHR: National Institute of Health Research
PCMHS: Primary Care Mental Health Service
PHQ-9: Patient health questionnaire
PTCI: Post-traumatic cognitions inventory
PTSD: Post-traumatic stress disorder
RCT: Randomised controlled trial
SAE: Serious adverse event
SARCS: Sexual Assault Referral Centres
TFCBT: Trauma focused cognitive behavioural therapy
TFPT: Trauma focused psychological therapy
TSC: Trial steering committee
TSQ: Trauma screening questionnaire
WSAS: Work and social adjustment scale
